# Psychosis Induced by Methylphenidate in Children and Young Patients With Attention-Deficit Hyperactivity Disorder

**DOI:** 10.7759/cureus.34299

**Published:** 2023-01-28

**Authors:** Khadija Pasha, Salomi Paul, Muhammad S Abbas, Sondos T Nassar, Tasniem Tasha, Anjali Desai, Anjana Bajgain, Asna Ali, Chandrani Dutta, Abeer O Elshaikh

**Affiliations:** 1 Pediatric, California Institute of Behavioral Neurosciences & Psychology, Fairfield, USA; 2 Medicine, California Institute of Behavioral Neurosciences & Psychology, Fairfield, USA; 3 Psychiatry, California Institute of Behavioral Neurosciences & Psychology, Fairfield, USA; 4 Medicine and Surgery, Jordan University of Science and Technology, Amman, JOR; 5 Internal Medicine, Rajshahi Medical College, Rajshahi, BGD; 6 Internal Medicine, California Institute of Behavioral Neurosciences & Psychology, Fairfield, USA; 7 Research and Academic Affairs, Larkin Community Hospital, South Miami, USA; 8 Family Medicine, California Institute of Behavioral Neurosciences & Psychology, Fairfield, USA; 9 Internal Medicine/Family Medicine, California Institute of Behavioral Neurosciences & Psychology, Fairfield, USA

**Keywords:** adhd, dosage of mph, treatment of adhd, delusions, hallucinations, amphetamine, schizophrenia, psychosis, methylphenidate, psychostimulants

## Abstract

Attention-deficit hyperactivity disorder (ADHD) is one of the most common neurodevelopmental disorders diagnosed in children of this era. ADHD in children and adults is challenging but highly manageable. Children with ADHD cannot focus, are hyperactive, and appear withdrawn. These symptoms make them endure difficulties in learning and create academic challenges. Methylphenidate (MPH) is one of the psychostimulants used as a first-line therapy for ADHD.

In this literature review, we have gathered information that describes the evidence of psychotic symptoms in children and young patients with ADHD as a side effect of MPH. We used articles from the National Library of Medicine (PubMed) and Google Scholar to gather the relevant information. Our findings concluded that MPH can increase the risk of psychosis, particularly when taken in high doses. It is still unclear whether the psychotic range of symptoms occurred due to an increased dopamine (DA) level caused by MPH or as a predominant feature of ADHD or if there was another comorbid feature in the history of the patient that led to psychosis. However, a necessary step for every medical practitioner prescribing psychostimulants is that they enlighten the patient and caregiver that this rare but threatening side effect is a possibility.

## Introduction and background

Attention-deficit hyperactivity disorder (ADHD) has always been a subject of interest for researchers [1]. The estimated prevalence of ADHD is between 5% and 10% in American children and 2.8% and 5.2% in adults [2]. Boys are diagnosed two to four times more often than girls [3]. About 3% of children treated with methylphenidate (MPH) are reported to experience serious adverse events such as psychosis and mood disorders [3]. The three types of ADHD are primarily hyperactive and impulsive, primarily distracted, or inattentive and combined [4]. Each presentation is distinguished by a set of behavioral symptoms described in the Diagnostic and Statistical Manual of Mental Disorders, Fifth edition (DSM-5) that physicians use to diagnose ADHD and its subtype [4]. The inattentive subtype is more common in girls [5]. According to DSM-5's diagnostic criteria, symptoms of ADHD must be present for more than six months in more than two settings such as school, home, and church [2]. People with ADHD often have significant dysfunction in academics and familial and social situations [6]. Adolescents with ADHD are at increased risk of school failure due to learning and language problems [6]. Other consequences related to ADHD include dangerous driving, impaired relationships with peers, criminal activity, and impulsive sexuality [6].

The prefrontal cortex (PFC) is an important area of ​​the brain that mediates cognitive and executive functions, such as working memory, sustained attention, inhibitory response control, and cognitive flexibility [7]. Delayed PFC maturation, frontal lobe circuit dysfunction, and frontal cortex hypoactivity are associated with ADHD patients [7]. MPH, a psychostimulant, acts as an indirect dopamine (DA) agonist and enhances neuronal signaling by inducing significant increases in extracellular levels of neurotransmitters in the synaptic cleft of the PFC and is, therefore, considered the first-line treatment for ADHD [8]. Psychosis refers to a strange psychological state, which is frequently described as related to a *lack of touch with reality* [9]. Psychosis is a multisymptom clinical syndrome, delusions, hallucinations, and thought disorders can be considered to be core clinical features [9].

The primary objective of this literature review is to examine the relevant data that shows a correlation between the use of MPH and psychosis as a side effect in children and young patients with ADHD. Databases such as *PubMed* and *Google Scholar* were utilized to search for English literature articles. MeSH terms such as ADHD, MPH, psychosis, schizophrenia, and psychostimulants were used to search for appropriate articles. Multiple design studies are used. Filters included free full-text articles and those published within the past 10 years (January 2013 to June 2022), with children and young patients aged 5 to 30 years. Out of 1,400 articles, 24 were shortlisted based on relevance to the topic of discussion.

## Review

Relationship between the new onset of psychosis and MPH in ADHD

**Figure 1:**
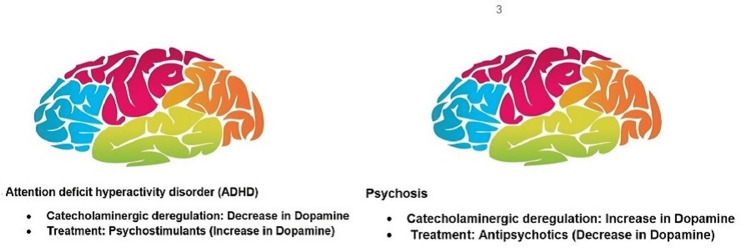
Pathophysiology of ADHD and psychosis. Figure credits: Khadija Pasha. ADHD, attention-deficit hyperactivity disorder

Stimulants such as MPH and amphetamine (AMP) are the most common pharmacological treatments for ADHD [10]. Although their mechanisms of action differ, the main effects of AMP and MPH on the central nervous system of the brain include the availability of catecholamines in striatal and cortical regions, as demonstrated in preclinical studies [11]. The most reported side effects associated with MPH are headaches, sleep disorders, malaise, abdominal pain, and loss of appetite [12]. In 2007, the Food and Drug Administration (FDA) ordered a change in the drug label for stimulants, based on new evidence of psychosis [13,14]. Psychosis is considered to be a DA excess disorder, treated with DA antagonists, whereas ADHD is considered to be a low-DA disorder, treated with DA agonists (Figure 1) [15].

Ramstad et al. evaluated the proof of psychotic signs and symptoms as a detrimental impact of MPH treatment in children and adolescents with ADHD [16]. Their article summarizes the applicable effects from the Cochrane systematic review using meta-analysis and trial sequential analysis on MPH remedy for ADHD that is posted elsewhere [16]. Based on a specific evaluation of present studies, Ramstad et al. recorded that there is not enough proof to judge whether MPH is related to remedy-emergent psychotic signs and symptoms [16]. However, psychotic signs and symptoms might develop in 1.1% to 2.5% of those being dealt with MPH, so clinicians ought to be alert to the opportunity that psychotic signs and symptoms might once in a while arise all through treatment with MPH [16].

Another meta-analysis of the nonrandomized clinical study conducted by Storebø et al. included a total of 2,345 patients who were categorized based on exposure to MPH into control and experimental groups [3]. Participants aged between 3 and 20 years were selected [3]. The result suggests that about one in a hundred patients treated with MPH can experience serious adverse effects such as cardiac disorders, psychotic disorders, and death [3]. Approximately 1.2% of the hundred patients discontinued MPH due to a serious adverse event [3]. Another point highlighted in this study describes the daily treatment range for MPH doses in children that ranged from 5 to 60 mg, one to three times daily, depending on the delivery system and method of administration [3]. Higher doses of MPH are more prone to side effects [3].

A cohort study conducted by Moran et al. included patients in the age group of 13 to 25 years, diagnosed with ADHD, and taking MPH between January 1, 2004, and September 30, 2015 [14]. The outcome showed a new onset of psychotic symptoms in one in 600 patients [14]. This study correlates the idea of psychosis with an increased level of DA caused by psychostimulants [14]. It mentions that psychostimulants inhibit DA transporters that induce DA release from neurons and facilitate DA reuptake into presynaptic terminals [14]. However, DA release from AMP is four times greater than MPH, although MPH is a more potent inhibitor of the DA transporter [14]. The study also mentions that the neurotransmission changes observed in primary psychosis are more persistent with AMP than by MPH [14].

Another cohort study conducted by Moran et al. selected 239 patients who had a history of psychotic disorders [17]. Out of these 239 patients, 113 had a history of prior exposure to prescription stimulants, which was associated with an earlier age of onset of psychosis [17]. Although they mention a hypothesis that states that the stimulant itself is not responsible for the earlier onset of psychosis, earlier presentation is due to other factors such as intellectual disability or a diagnosis of ADHD [17].

Cortese, in his literature review, suggests that some individuals are *insensitive* (rarely develop psychosis after exposure to stimulants) and some are *sensitive* (highly likely to develop psychosis after taking low doses of stimulants or without exposure to stimulants) [18]. This study does not define the predictability criteria to select patients who are vulnerable to psychosis but suggests an interesting notion that describes that those psychotic symptoms were not present when the drug was commenced by a psychiatrist as compared to a physician [18]. Professionals such as psychiatrists more readily detected prodromal psychotic features that increased the risk of stimulant-induced psychosis and avoided the prescription of MPH in such cases [18]. The analysis of the FDA data and case reports have shown that in 92% of patients, psychotic symptoms are short-lived and resolve after the discontinuation of the stimulant, even without treatment using antipsychotic medications [18].

Walichniewicz and Lew-Starowicz, in their case report, describe the case of a 31-year-old female patient who developed MPH-induced psychosis after taking MPH for almost 15 months [19]. She had a positive family history of schizophrenia [19]. This study highlights that psychosis is relatively rare, but it is potentially a serious side effect of the use of MPH [19]. However, studies have not been able to establish a definite cause for this infrequent adverse outcome. Careful evaluation should be executed before prescribing MPH for the patient’s safety, including clinical examination and both medical history and family history [19]. Psychostimulants should be used with caution in children and adolescents with a family history of bipolar disorder and schizophrenia [13]. According to a literature review by Runde, psychostimulants may accelerate mood and psychotic symptoms in adolescents who already have a genetic predisposition to these mental disorders [13].

Man et al. conducted a study using electronic medical records on the Clinical Data Analysis and Reporting System (2001-2014) [20]. His team observed 20,586 patients who were taking MPH [20]. Among those, 103 patients had psychotic events [20]. The mean age of starting the drug was around six years, because MPH is not considered safe for children aged below six years, and the mean period for a follow-up was around 10 years [20]. Out of 103 patients, 76 had ADHD as a preliminary diagnosis [20]. Of the 103 psychotic events, 78 occurred during the baseline period and 25 occurred during the MPH treatment period [20]. This study does not support the theory that MPH increases the risk of psychotic events [20]. However, it shows that the risk of such an event is higher before the first prescription of MPH [20]. This may be due to the association between behavioral and attentional symptoms with psychotic events that led to the diagnosis of psychosis and initiation of treatment with MPH [20].

Management of psychosis after MPH use and treatment of ADHD after an episode of psychosis

In his case report, Güneş describes the case of a 16-year-old boy who had symptoms of ADHD and mild intellectual instability [21]. The patient was started on a sustained release of MPH of 20 mg/day [21]. One week after the use of MPH, he was admitted to the emergency department with complaints of mania, speaking louder and appearing more energetic than usual [21]. His psychiatric assessment revealed euphoria, grandiosity, and discordant effects [21]. MPH was discontinued, but his symptoms did not resolve [21]. Valproic acid 1,000 mg/day and risperidone 2 mg/day were commenced, and his symptoms began to diminish within two weeks [21]. Within three months, he improved significantly [21]. This case report suggests that therapeutic doses of MPH can cause a psychotic range of symptoms in rare circumstances [21]. Symptoms usually began after starting medication or shortly after increasing the dose [21].

Beckmann et al. described MPH as a cause of psychosis in ADHD patients, particularly when taken in high doses [22]. If a patient develops MPH-induced psychosis, then the medication should be stopped [22]. If continued, pharmacotherapy is needed to treat an underlying psychiatric or medical condition, and an alternative medication should be prescribed if possible [22].

In her review article, Hechtman emphasizes the importance of slowly titrating stimulant medications such as MPH and informing the patient about the possibility of developing this side effect [23]. If the patient happens to develop a range of psychotic symptoms, antipsychotic medications such as risperidone can be used as an additional treatment [23].

In a case report, Gable and Depry outlined a 26-year-old patient with ADHD who required hospital admission after taking the prescribed dose of a stimulant [24]. The report points out that patients taking stimulants to treat ADHD may be at risk of developing psychosis [24]. Until the underlying risk factors become apparent, all practitioners need to have a complete psychiatric and family history of schizophrenia before prescribing the medication [24]. It is also important for patients to carefully monitor behavioral changes at the start of treatment and during induction while emphasizing the importance of taking prescribed medications to avoid the development of psychosis [24].

Limitations

The current conclusions have been drawn from accessible studies available on PubMed and Google Scholar databases published in English literature. The data presented was not representative of all age groups. We have included articles that were published in the past 10 years. Conflicting findings have been presented in various studies. The exact dose of MPH responsible for adverse outcomes has not been clarified.

## Conclusions

We studied the relationship between psychosis and MPH in children and young patients with ADHD. We have found a considerable amount of evidence that suggests prescribing MPH puts the patient at risk of developing a rare side effect of psychosis, which can lead to hallucinations and delusions, whereas some studies did not obtain the same result. However, physicians should be aware that psychotic symptoms can occur during treatment with MPH. It is their responsibility to educate patients and their caretakers about this rare but serious side effect.

Further research in children and young patients with ADHD is needed to identify the side effects of MPH to provide data that is representative of different age groups. A more detailed study with larger samples should be performed that provides statistically significant results. Some questions remain unanswered such as the exact dose or range of doses of MPH that leads to psychosis. The relationship between the family history of psychosis and the development of psychotic features by MPH should also be investigated further.

## References

[1] Krinzinger H, Hall CL, Groom MJ (2019). Neurological and psychiatric adverse effects of long-term methylphenidate treatment in ADHD: a map of the current evidence. Neurosci Biobehav Rev.

[2] Tucker JE (2021). Prescription stimulant-induced neurotoxicity: mechanisms, outcomes, and relevance to ADHD. Mich J Med.

[3] Storebø OJ, Pedersen N, Ramstad E (2018). Methylphenidate for attention deficit hyperactivity disorder (ADHD) in children and adolescents - assessment of adverse events in non-randomised studies. Cochrane Database Syst Rev.

[4] Epstein JN, Loren RE (2013). Changes in the definition of ADHD in DSM-5: subtle but important. Neuropsychiatry (London).

[5] Felt BT, Biermann B, Christner JG, Kochhar P, Harrison RV (2014). Diagnosis and management of ADHD in children. Am Fam Physician.

[6] Lakhan SE, Kirchgessner A (2012). Prescription stimulants in individuals with and without attention deficit hyperactivity disorder: misuse, cognitive impact, and adverse effects. Brain Behav.

[7] Cheng J, Xiong Z, Duffney LJ (2014). Methylphenidate exerts dose-dependent effects on glutamate receptors and behaviors. Biol Psychiatry.

[8] Hollis C, Chen Q, Chang Z (2019). Methylphenidate and the risk of psychosis in adolescents and young adults: a population-based cohort study. Lancet Psychiatry.

[9] Gaebel W, Zielasek J (2015). Focus on psychosis. Dialogues Clin Neurosci.

[10] Gough A, Morrison J (2016). Managing the comorbidity of schizophrenia and ADHD. J Psychiatry Neurosci.

[11] Faraone SV (2018). The pharmacology of amphetamine and methylphenidate: relevance to the neurobiology of attention-deficit/hyperactivity disorder and other psychiatric comorbidities. Neurosci Biobehav Rev.

[12] Patel V, Krishna AS, Lefevre C, Kaagaza M, Wittkamp M (2017). Methylphenidate overdose causing secondary polydipsia and severe hyponatremia in an 8-year-old boy. Pediatr Emerg Care.

[13] Runde CL (2018). Psychostimulants and psychosis. Nurs Capstones.

[14] Moran LV, Ongur D, Hsu J, Castro VM, Perlis RH, Schneeweiss S (2019). Psychosis with methylphenidate or amphetamine in patients with ADHD. N Engl J Med.

[15] Levy E, Traicu A, Iyer S, Malla A, Joober R (2015). Psychotic disorders comorbid with attention-deficit hyperactivity disorder: an important knowledge gap. Can J Psychiatry.

[16] Ramstad E, Storebø OJ, Gerner T (2018). Hallucinations and other psychotic symptoms in response to methylphenidate in children and adolescents with attention-deficit/hyperactivity disorder: a Cochrane systematic review with meta-analysis and trial sequential analysis(). Scand J Child Adolesc Psychiatr Psychol.

[17] Moran LV, Masters GA, Pingali S, Cohen BM, Liebson E, Rajarethinam RP, Ongur D (2015). Prescription stimulant use is associated with earlier onset of psychosis. J Psychiatr Res.

[18] Cortese S (2019). Psychosis during attention deficit-hyperactivity disorder treatment with stimulants. N Engl J Med.

[19] Walichniewicz P, Lew-Starowicz M (20211). Methylphenidate-induced psychosis in a young antipsychotic-naïve female patient. Wiedza Medyczna.

[20] Man KK, Coghill D, Chan EW (2016). Methylphenidate and the risk of psychotic disorders and hallucinations in children and adolescents in a large health system. Transl Psychiatry.

[21] Güneş S (2017). Manic symptoms due to modified-release methylphenidate use: an
adolescent case. Yeni Symp.

[22] Beckmann D, Lowman KL, Nargiso J, McKowen J, Watt L, Yule AM (2020). Substance-induced psychosis in youth. Child Adolesc Psychiatr Clin N Am.

[23] Hechtman L (2019). ADHD medication treatment and risk of psychosis. Lancet Psychiatry.

[24] Gable M, Depry D (2015). Psychosis requiring hospitalization in an adult ADHD patient on a therapeutic stimulant: a case report and review of treatment alternatives. Int J Clin Case Stud.

